# Synergizing Exchangeable Fluorophore Labels for Multitarget STED Microscopy

**DOI:** 10.1021/acsnano.2c07212

**Published:** 2022-10-12

**Authors:** Marius Glogger, Dongni Wang, Julian Kompa, Ashwin Balakrishnan, Julien Hiblot, Hans-Dieter Barth, Kai Johnsson, Mike Heilemann

**Affiliations:** † Institute of Physical and Theoretical Chemistry, Goethe University Frankfurt, Max-von-Laue Str. 7, 60438 Frankfurt, Germany; ‡ Department of Chemical Biology, Max Planck Institute for Medical Research, Jahnstrasse 29, 69120 Heidelberg, Germany; § Institute of Chemical Sciences and Engineering (ISIC), École Polytechnique Fédérale de Lausanne (EPFL), 1015 Lausanne, Switzerland

**Keywords:** Super-resolution microscopy, DNA-PAINT, STED, exchangeable fluorophores, multicolor imaging, live-cell imaging, Halo-Tag

## Abstract

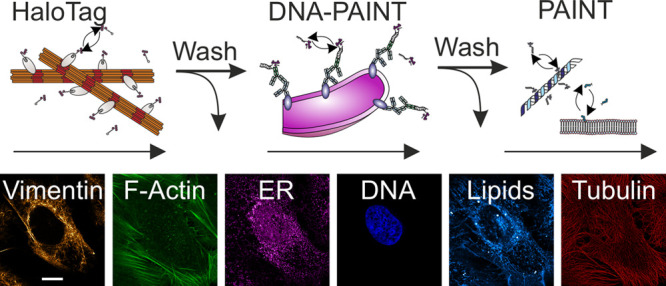

Investigating the interplay of cellular proteins with optical microscopy requires multitarget labeling. Spectral multiplexing using high-affinity or covalent labels is limited in the number of fluorophores that can be discriminated in a single imaging experiment. Advanced microscopy methods such as STED microscopy additionally demand balanced excitation, depletion, and emission wavelengths for all fluorophores, further reducing multiplexing capabilities. Noncovalent, weak-affinity labels bypass this “spectral barrier” through label exchange and sequential imaging of different targets. Here, we combine exchangeable HaloTag ligands, weak-affinity DNA hybridization, and hydrophophic and protein–peptide interactions to increase labeling flexibility and demonstrate six-target STED microscopy in single cells. We further show that exchangeable labels reduce photobleaching as well as facilitate long acquisition times and multicolor live-cell and high-fidelity 3D STED microscopy. The synergy of different types of exchangeable labels increases the multiplexing capabilities in fluorescence microscopy, and by that, the information content of microscopy images.

## Introduction

Stimulated emission depletion (STED) microscopy is a super-resolution microscopy technique with theoretically unlimited spatial resolution.^1^ In combination with well-established fluorescence labeling protocols, STED is a powerful tool in optical structural cell biology. As with many high-performance microscopy methods, STED microscopy demands for comparably high laser intensities, which may lead to photodestruction of covalently bound fluorophores and subsequent signal loss. As a consequence of photobleaching, long acquisition times in live-cell experiments as well as imaging thick samples in 3D with high fidelity is challenging. Attempts to reduce fluorophore photobleaching include adaptive illumination schemes,^2^ the development of photostable fluorophores,^3^ or the application of fluorescent proteins with multiple off-states.^4^ In addition, the number of cellular structures and proteins that can be imaged within the same cell is limited due to spectral overlap of fluorophore absorption and emission spectra.^5^ Both challenges can be addressed with weak-affinity, noncovalent fluorophore labels that transiently bind to a target structure and are continuously replaced by intact fluorophores from a large buffer reservoir. These “exchangeable” labels provide an elegant way to bypass photobleaching and facilitate multiplexing and 3D imaging.^6−8^ Originally introduced in the single-molecule imaging method termed point accumulation for imaging in nanoscale topography (PAINT^9^) using the hydrophobic dye Nile Red, the concept was generalized by the application of specific fluorescent ligands (uPAINT^10^) and was further extended to weak-affinity DNA–DNA hybridization (DNA-PAINT^11−13^), peptide fragments (IRIS^14^), peptide–peptide interactions,^15^ or protein-targeting oligonucleotides (aptamers^16^). HaloTag7 (HT7) is a self-labeling protein that covalently reacts with biorthogonal ligands (HTLs^17^). Recently introduced exchangeable HT7 ligands (xHTLs^18^) are cell membrane-permeable fluorescent ligands for super resolution microscopy, representing a powerful alternative for live-cell STED studies. xHTLs are C_4_ or C_5_ derivatives of covalent HTLs (Dye-PEG_2_-C_6_-Cl, chloroalkane) substituted with methylsulfonamide (Dye-PEG_2_-C_5_-methylsulfonamide (S5)),trifluoromethylsulfonamide (Dye-PEG_2_-C_5_-trifluoromethylsulfonamide (T4)) or hydroxy (Dye-PEG_2_-C_4/5_-hydroxy (Hy4/Hy5)) and conjugated to rhodamines (SiR and JF_635_ in this work). These modifications introduce exchangeability to HT7 with dissociation constants in the nanomolar range and fast binding kinetics. In addition, xHTLs can be fluorogenic, increasing fluorescence intensity (with a factor of ∼1.3–10) upon binding to HT7. Their exchangeable nature combined with their fluorogenicity makes them highly versatile as a fluorescent labeling system.^18^

Here we present sequential multimethod labeling combining the concepts of DNA-PAINT, PAINT/IRIS, and HaloTag-based PAINT (HT-PAINT) and image six different structures in a single eukaryotic cell using STED microscopy. We report imaging cellular structures with high spatial resolution and demonstrate reduced photobleaching compared to covalent labels. We further demonstrate the applicability of our cross-method labeling approach for multitarget live-cell and 3D imaging.

## Results and Discussion

**Figure 1:**
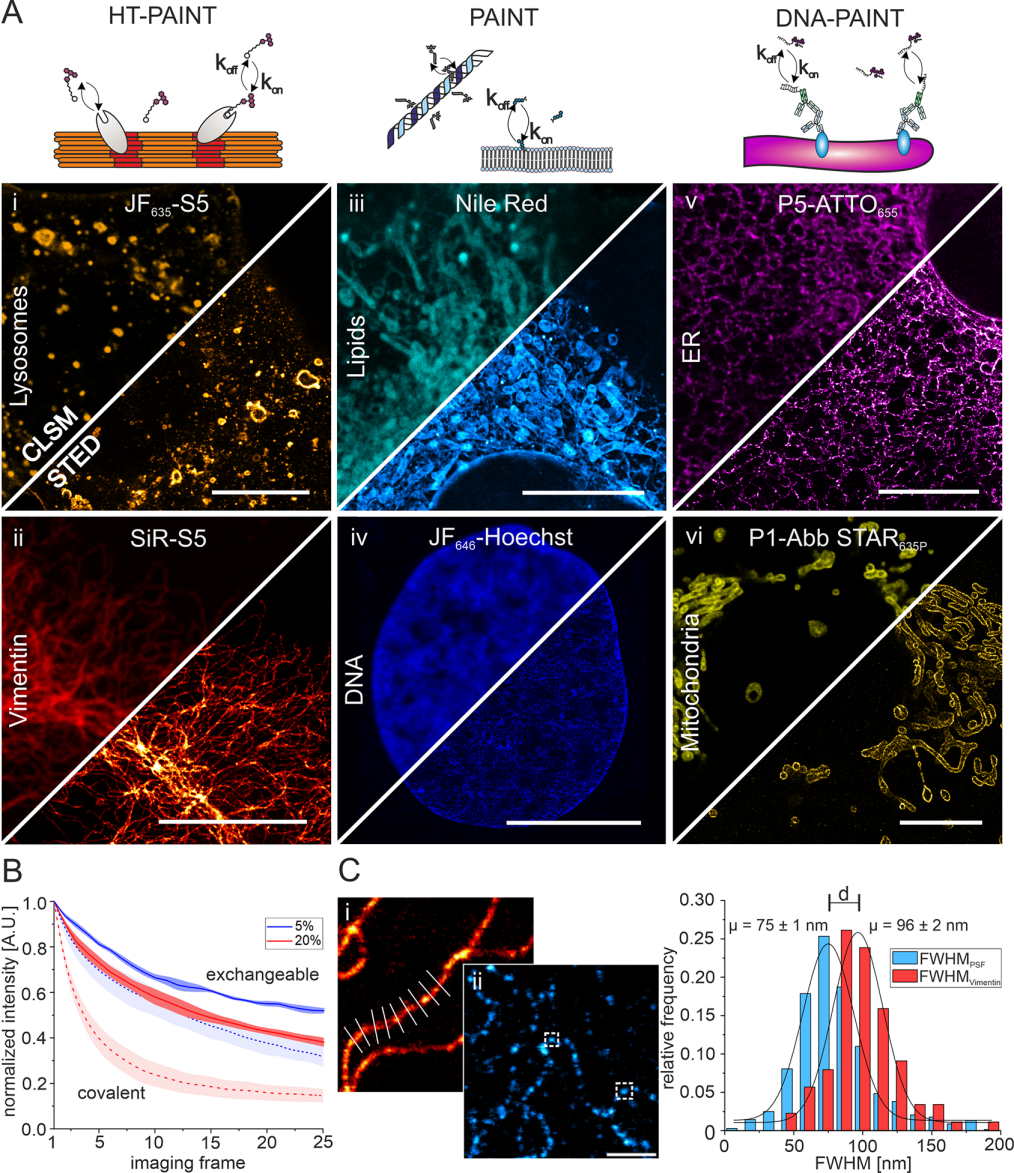
Exchangeable fluorescent labels for CLSM and STED microscopy of various cellular structures. (A) Illustration of the principle of transient ligand binding to target structures in HT-PAINT, PAINT, and DNA-PAINT (upper panel). Lower panels show CLSM and STED images using nonorthogonal xHTLs (LamP1-HT7 (i), 300 nM JF_635_-S5, gold; vimentin-HT7 (ii), 300 nM SiR-S5, red hot), PAINT labels (500 nM Nile Red (iii), cyan hot; 300 nM JF_646_-Hoechst (iv), blue), and DNA-PAINT labels (KDEL-antibody (v), 300 nM P5-ATTO_655_, magenta hot; TOM20-antibody (vi), 300 nM P1-Abb STAR_635P_, yellow). All scale bars in (i–vi) are 10 μm. (B) Comparison of fluorescence signal versus time recorded in CLSM mode for vimentin structures in U2OS cells using exchangeable (JF_635_-S5) or covalent HaloTag ligands (JF_635_-HTL) at different laser intensities (*N*_cells_ = 3–5). (C) Quantification of the resolution in STED images. The optical resolution was determined from the intensity profile perpendicular to continuous vimentin-HT7 structures ((i), 500 nM SiR-S4, red hot) or the full-width at half-maximum (fwhm) of individual vimentin-HT7 spots ((ii), 100 nM JF_635_-S5, cyan hot). Scale bar is 1 μm. Shown is the relative frequency distribution of the determined fwhm of individual spots (light blue, *n* = 691) and intensity profiles (red, *n* = 88).

We first tested various weak-affinity, exchangeable fluorophore labels by targeting and imaging different cellular structures individually with confocal laser scanning microscopy (CLSM) and STED microscopy, including xHTLs, PAINT/IRIS labels, and DNA-PAINT labels. Next to previously introduced xHTLs S5 and T4,^18^ we further synthesized and tested another exchangeable derivative (SiR-C_4_-methylsulfonamide (S4)) for its applicability in STED microscopy (SI Methods, SI Spectrum 1). Sufficiently high ligand concentrations (100–500 nM) in the imaging buffer ensured a saturation of targets and thus quasipermanent labeling and allowed imaging various structures with high quality (Figure 1A). Imaging with super-resolution STED microscopy visualized structural features obscured in diffraction-limited confocal microscopy (SI Figure 1). We next evaluated the applicability of our approach in confocal time-course measurements. DNA-PAINT and PAINT labels were previously shown to reduce photobleaching and facilitate time-lapse and volumetric imaging in CLSM and STED configurations.^7,19^ To evaluate whether this applies to xHTLs, we determined the degree of photobleaching using time-lapse confocal microscopy of endogenously tagged vimentin-HT7 in U2OS cells.^20^ We compared the HT7 covalent labeling (JF_635_-HTL) to its fluorogenic xHTL counterpart (JF_635_-S5^18^) at two different irradiation intensities (Figure 1B). For this purpose, we applied an analysis pipeline that generates a binary mask on drift-corrected time-series for image segmentation and recorded signal and background over time (SI Methods).^7^ Intensity–time traces showed that the signal intensity decreased strongly for the covalent labels over the first 25 consecutive frames, whereas we observed a moderate degree of photobleaching for exchangeable labels (Figure 1B, SI Figure 2A). These findings are in good agreement with intensity–time trace analysis for DNA-PAINT and PAINT labels (SI Figure 2B) and demonstrate the applicability of xHTLs for multiframe time-lapse confocal microscopy. Next, for DNA-PAINT labels, we reduced the DNA–DNA hybridization time by supplementing the imaging buffer with ethylene carbonate (as demonstrated in DNA-PAINT-ERS^21^). We measured a further reduced degree of photobleaching (SI Figure 2B) possibly caused by faster replacement of photodamaged labels. We imagine that faster exchange rates for xHTL labels could have similar effects.

Next, we determined the spatial resolution of STED images from cells labeled with xHTLs. Making use of the inherent flexibility of transient labels to adjust the label density within the sample and control target saturation, we decided to determine the resolution in different ways. First, we used high label densities to achieve quasipermanent labeling and imaged continuous vimentin filaments. We then determined the full-width at half-maximum (fwhm) of the structure by fitting the intensity profile perpendicular to the filaments with a Gaussian function. Full labeling of vimentin filaments resulted in an average fwhm of 96 ± 2 nm (mean, s.d.) (Figure 1C). Second, we imaged sparsely labeled vimentin-HT7 structures followed by the determination of the fwhm of individual fluorescent spots. From the distribution of individual fluorescent spots, we measure an average fwhm of 75 ± 1 nm and as small as <40 nm (Figure 1C). The increased average fwhm value in the case of full vimentin labeling is in good agreement with our single-spot analysis considering the width of single vimentin filaments (10 nm^22^) and additional linkage error introduced by the HaloTag system (PDB-ID: 7ZJ0). We further evaluated the spatial resolution by analyzing the intensity profiles of vimentin structures at intersection points (SI Figure 3) and show the optical separation of single filaments closer than 86 nm. Additionally, we used Fourier ring correlation (FRC) analysis^23^ to compare the resolution of vimentin structures imaged with either exchangeable or covalent HT7 ligands. In both cases, we determined a spatial resolution of better than 70 nm (SI Figure 4), demonstrating the applicability of xHTLs for STED microscopy. The spatial resolution was slightly better in the case of covalent labeling, in line with increased signal-to-background ratios (SBR) and signal-to-noise ratios (SNR). This might arise from residual fluorescence emission of free xHTLs in the imaging buffer that contribute to background fluorescence (SI Figure 4A). Taken together, the experiments demonstrate that xHTLs enable STED imaging with subdiffraction spatial resolution. The main goal of this study was to demonstrate that multiple types of exchangeable labels can be combined to multitarget STED microscopy. For this purpose, we operated a standard commercial STED microscope (Experimental Methods). Several experimental strategies were reported to improve the spatial resolution in STED microscopy^24−27^ and may be implemented in this workflow if available.

**Figure 2:**
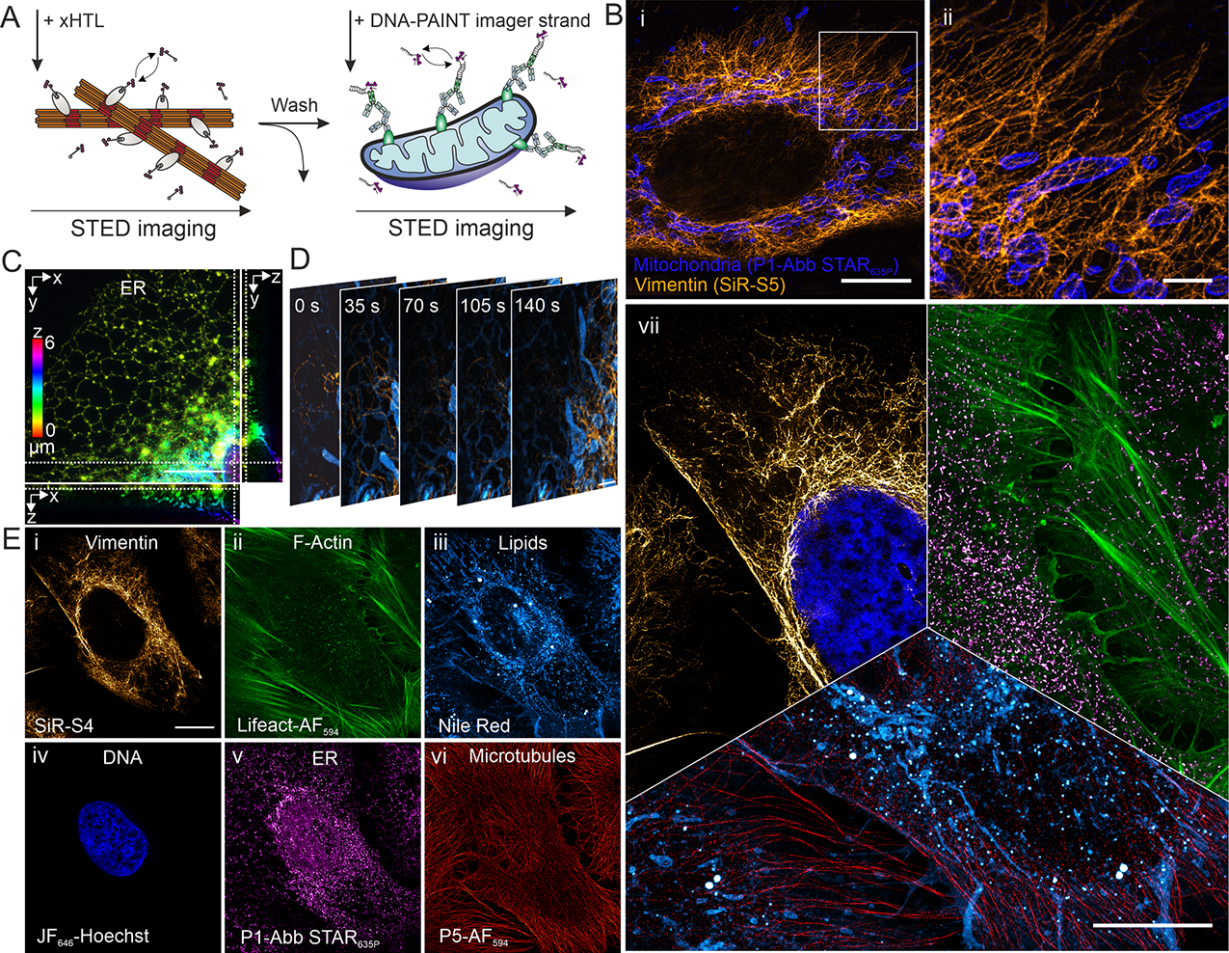
Multitarget, multicolor, and multilabeling method STED microscopy in single cells using exchangeable fluorescent labels. (A) Schematic illustration of the principle of ligand exchange in multilabeling method STED microscopy. Target specific and transient binding ligands for HT-PAINT and DNA-PAINT are exchanged between imaging rounds. (B) Cross-method labeling and two-target STED microscopy in a single cell. (i) STED image of vimentin-HT7 labeled with xHTLs (300 nM SiR-S5, orange hot) and mitochondria (TOM20) labeled with a DNA-PAINT imager strand (300 nM P1-Abb STAR_635P_, blue). White box indicates magnified region shown in (ii). Scale bars are 10 μm (i) and 2 μm (ii). (C) 3D-STED imaging of the ER located fusion protein CalR-HT7-KDEL in a U2OS cell. The cell was labeled using the xHTL SiR-S5 (300 nM). Image shows deconvoluted 3D-STED z-projection and corresponding ortho-slices. Scale bar z-projection is 5 μm. (D) Live-cell two-color STED microscopy using exchangeable fluorescent and cell membrane-permeable ligands. U2OS cells were labeled for the endomembrane system (500 nM Nile Red, cyan hot) and vimentin-HT7 (500 nM JF_635_-S5, orange hot). Scale bar is 2 μm. (E) Six-target super-resolution STED image of various structures in a single U2OS cell. Cross-method labeling STED imaging was performed via HT-PAINT ((i), vimentin, 300 nM SiR-S4), IRIS/PAINT ((ii–iv), F-actin, 1 μM Lifeact-AF_594_, 300 nM; membranes, 300 nM Nile Red; chromosomal DNA, 300 nM JF_646_-Hoechst), and DNA-PAINT ((v,vi), ER, 500 nM P1-Abb STAR_635p_; microtubules, 500 nM P5-AF_594_). Images were aligned using fiducial markers. Scale bar is 10 μm. (vi) Six-target STED image in a single cell. Overlay of sequentially recorded STED images from multimethod labeling approaches (i–vi). Scale bar is 10 μm.

We next explored whether a combination of various types of exchangeable labels would enable multicolor imaging of multiple structures in the same cell. We first validated the cross-method labeling compatibility by sequential labeling and imaging targets with xHTLs and DNA-PAINT labels with similar spectral properties in single cells (Figure 2A,B). Exchange of the fluorescent labels between imaging cycles was conducted via manual pipetting or assisted by a microfluidic system and completed within 5–9 min. The exchange-based approach facilitated high fidelity and residual-free imaging of individual vimentin and mitochondria. Next, we explored the applicability of xHTLs for 3D STED microscopy. We first labeled chemically fixed U2OS cells expressing CalR-HT7-KDEL (endoplasmic reticulum, ER) using the xHTL SiR-S5 and recorded z-stack images in 3D STED mode. Volumetric rendering of deconvoluted images allowed high fidelity reconstruction of the ER (Figure 2C, Supplementary Video 1). Further, we used cross-method labeling and sequential STED microscopy in one spectral channel for 3D imaging of chromosomal DNA (JF_646_-Hoechst^28^) and the ER (SiR-S5) in the CalR-HT7-KDEL cell line. Fiducial marker-based image alignment in all dimensions and volumetric rendering allowed two-target 3D STED microscopy (Supplementary Video 2). Encouraged by these findings, we applied our cross-method labeling approach for two-color live-cell STED microscopy. We concurrently labeled the endomembrane system and vimentin-HT7 using the membrane-permeable label Nile Red and JF_635_-S5, respectively. Simultaneous excitation with 561 and 640 nm and depletion at 775 nm facilitated two-color live-cell STED microscopy and allowed following the dynamics of both structures (Figure 2D, Supplementary Video 3). Finally, we combined HT-PAINT, DNA-PAINT, and PAINT/IRIS labeling methods in a single cell and achieved STED imaging of six different structures (Figure 2E). We used three far-red (SiR, JF_646_, and Abberior STAR_635P_ (Abb STAR _635P_)) and two red-emitting fluorescent dyes (AlexaFluor_594_ (AF_594_)_,_ Nile Red) and exchanged the fluorophore labels between STED imaging cycles by washing steps within 5–9 min. Lateral shift caused by the exchange was corrected using fiducial markers. This procedure facilitated precise alignment of all imaging channels and the reconstruction of highly multiplexed STED images of a single cell.

## Conclusions

In summary, we demonstrate six-target STED microscopy in single cells using exchangeable fluorophore labels. This is achieved by combining different types of exchangeable labels, employing weak-affinity DNA hybridization, exchangeable HaloTag ligands, a protein-targeting peptide, a membrane label, and a DNA binder, all sharing the feature of transient target binding. We demonstrate the ability of these labeling approaches to substantially reduce photobleaching and the compatibility to STED imaging of cellular structures with subdiffraction spatial resolution. Further improvements in spatial resolution can be achieved by tuning STED imaging parameters and ligand binding kinetics as demonstrated for DNA hybridization.^6^

Sequential labeling with exchangeable labels is the key to enable many-target STED imaging in fixed cells, as it bypasses the limitations of covalent labels in STED microscopy caused by spectral overlap and balancing excitation, emission, and depletion wavelengths. The approach is scalable, and labeling and imaging of even more targets is possible by implementing, e.g., orthogonal xHTLs,^18^ increasing the number of DNA-barcoded labels, by integrating orthogonal protein labels,^8^ or by implementing other weak-affinity labels^29−31^. Given the availability of highly specific target-ligand pairs in molecular biology, we envisage many more types of exchangeable labels that could turn compatible with this approach and further expand the multiplexing. Other attempts to overcome the spectral barrier in fluorescence microscopy applied fluorescence lifetime imaging (FLIM^32^), spectral unmixing,^33^ or a combination of both (sFLIM^34^). These approaches can be combined with the use of exchangeable labels with different spectral properties and potentially help to minimize the number of washing steps by allowing pairwise imaging of labels.

Exchangeable labels continuously bind and unbind to their target, which leads to a replenishment of eventually photobleached fluorophores with intact fluorophores from the imaging buffer. This leads to an increased signal over time compared to covalent labels, which is beneficial for the image quality in 3D STED microscopy. In the case of live-cell compatible exchangeable labels, long-time live-cell imaging is enabled.^6^

We envisage application of exchangeable labels for complex multiprotein studies in single cells, targeting, e.g., spatial patterns of proteins and interactions in organelles at the nanoscale with high spatial resolution. Substituting covalent labels by exchangeable labels and using combinations of different labeling methods overcome the spectral barrier and facilitate studying a multitude of proteins in the same cell. This concept will elevate fluorescence microscopy into an optical omics method for structural cell biology.

## Experimental Methods

### Molecular and Cell Biology

#### Cell Culture

Eukaryotic cells (U2OS or U2OS vimentin-HT7) were cultured in T-75 flasks (Greiner) at 37 °C and 5% CO_2_ in Dulbecco’s Modified Eagle Medium (DMEM)/F-12 (Gibco, Thermo Fisher, USA) containing 10% (v/v) fetal bovine serum (FBS) (Corning, USA), 1% penicillin-streptomycin (w/v) (Gibco, ThermoFisher, USA), and 1% GlutaMAX (v/v) (Gibco, USA). Cells were passaged every 3–4 days using PBS and trypsin treatment and tested for mycoplasma contamination on regular intervals. Cells used for fixed-cell imaging were seeded on fibronectin-coated (Sigma-Aldrich, Germany, 0.1% (v/v) fibronectin human plasma for 30 min) microscopy chambers (μ-slides VI 0.4, Ibidi, 3 × 10^4^ cells/channel) 24 h prior to fixation. For live-cell microscopy, cells were seeded on fibronectin-coated eight-well chamber slides (Sarstedt, Germany, 1.5 × 10^4^ cells/well) for 24 h. Prior to imaging, the cells were washed with live-cell imaging solution (LCIS, ThermoFisher) and temperature adjusted to reduce lateral and axial drift.

#### Plasmids

All cell lines transiently expressing HT7 fusion proteins and stable cell lines used in this study are described in detail by Kompa et al.^18^ For STED microscopy of lysosomes (LamP1-HT7) and the endoplasmic reticulum (CalR-HT7-KDEL) using xHTLs, cells were transiently transfected with plasmids pCDNA5/FRT/TO_TOM20-dHaloTag7_T2A_LamP1-HaloTag7 (Addgene #187078) and pCDNA5/FRT/TO_TOM20-dHaloTag7_T2A_CalR-HaloTag7-KDEL (Addgene # 187079), respectively. For this purpose, 3 × 10^4^ U2OS cells were seeded on fibronectin-coated microscopy chambers. After 24 h of incubation (37 °C, 5% CO_2_), cells were transfected using Lipofectamine 3000 transfection reagent (Gibco, Thermo Fisher, USA). Briefly, 0.31 μL of Lipofectamine 3000 was diluted in 10.42 μL of OptiMEM medium (Gibco, Thermo Fisher, USA), and 105 ng of vector DNA was diluted in 10.42 μL of OptiMEM medium with 0.42 μL of P3000 reagent (Gibco, Thermo Fisher, USA). Diluted DNA solution was added to Lipofectamine diluent in a 1:1 ratio and incubated for 15 min at RT. After the DNA–lipid complex was added, cells were further incubated for 48 h at 37 °C and 5% CO_2_.

### Microscopy

#### Sample Preparation

##### Cell Fixation and DNA-PAINT Labeling

U2OS cells were chemically fixed with prewarmed (37 °C) 4% PFA (Gibco, Thermo Fisher, USA) with or without 0.1% GA (Sigma-Aldrich Chemie GmbH, Germany) in PBS (Gibco, Thermo Fisher, USA) and incubated (37 °C 5% CO_2_) for 20 min. After washing samples with PBS, cells were permeabilized and blocked for 1 h at RT using permeabilization/blocking buffer (PB, 3% IgG-free BSA, 0.1–0.2% saponin, PBS). Subsequently, primary antibodies (Supplementary Table 1) were diluted in PB, added to the chambers, and incubated for 90 min at RT. Excess primary antibody was removed by washing the sample thrice with PBS. Custom DNA docking strand-labeled secondary antibodies (Supplementary Table 1) were diluted in PB and incubated for 90 min at RT. After excess secondary antibodies were removed by washing with PBS, the samples were postfixed with 4% PFA for 10 min at RT and finally washed thrice with PBS. Prior to CLSM and STED imaging, DNA-PAINT imager strands (Supplemesntary Table 2) were diluted in imaging buffer (500 mM NaCl in PBS, pH 8.3) and added to the chambers.

##### Cell Fixation and Labeling in HT-PAINT and PAINT

U2OS mother cell lines or U2OS cells expressing HT7 fusion proteins were chemically fixed with prewarmed (37 °C) 4% PFA and 0.1% GA in PBS and incubated (37 °C, 5% CO_2_) for 20 min, followed by washing with PBS. Prior to CLSM and STED microscopy, exchangeable labels were diluted in PBS and added to the chambers for labeling of vimentin, lysosomes, ER (all using xHTLs), lipids (Nile Red), F-actin (LifeAct-AF_594_), and chromosomal DNA (JF_646_-Hoechst).

#### STED Microscopy

STED and confocal laser scanning microscopy were performed on the Abberior STED Expert Line microscope (Abberior Instruments, Göttingen, Germany) composed of an Olympus IX83 inverted microscope (Olympus, Japan) with a UPLXAPO 60× NA 1.42 oil immersion objective (Olympus, Japan) and operated by the Imspector software (v16.3.15507; Abberior Instruments, Göttingen, Germany). For fixed-cell imaging, fluorophores were excited by 561 nm laser light with a typical spectral window of 580–690 nm or by 640 nm laser light with a typical spectral window of 650–760 nm. For simultaneous dual color live-cell imaging, two spectral windows of typically 580–630 and 650–760 nm were applied under 561 and 640 nm laser excitation, respectively. The stimulated emission was performed with a 775 nm pulsed laser (Abberior Instruments, Göttingen, Germany), which is far from the spectral window of the orange channel (580–690 nm, excited by 561 nm laser) compared with the red channel (650–760 nm, excited by 640 nm laser). To compensate for this, the STED laser intensity was increased accordingly for the orange channel to ensure imaging quality. The fluorescence emission was recorded in line sequential mode and collected on avalanche photodiodes (APDs) using a gating of 0.75–8 ns. The pinhole was set to 0.61–0.71 AU for STED and CLSM. The pixel size was set to 20 nm for 2D-STED microscopy and 30/40 nm for 3D-STED microscopy. For volumetric imaging in 3D-STED mode, the z-stack step size was set to 40 nm for single-color imaging and 100 nm for dual color imaging. Line accumulation was set to 10–15, and dwell times were set to 3–15 μs, if not stated otherwise. Exchangeable labels and applied concentrations for experiments are provided in Supplementary Table 3. Detailed imaging settings for each measurement are listed in Supplementary Table 4.

##### Intensity Time Traces of Covalent and Exchangeable HaloTag Ligands

For intensity time trace analysis, covalent (JF_635_-HTL) and exchangeable HaloTag ligands (JF_635_-S5) were diluted to 100 nM in PBS. For covalent labeling, diluted JF_635_-HTL was added to the fixed U2OS vimentin-HT7 cells and incubated for 30 min at RT. Afterward, cells were washed thrice with PBS. Intensity time traces were recorded in confocal laser scanning mode. A total of 3–5 positions (approximately 10 × 10 μm^2^) were selected for each excitation intensity, and 25 consecutive frames were recorded using the following imaging parametesr: pixel size 20 nm, dwell time 2 μs, line accumulation 10, pinhole 0.71 AU for the xHTL JF_635_-S5 and 1.0 AU for covalent JF_635_ HTL.

##### Multitarget Fixed-Cell STED Microscopy

For multitarget STED imaging, fluorescent labels were exchanged between imaging rounds in sequence either by manual pipetting or assisted by a microfluidics system (Bruker, USA) controlled by Vutara’s SRX software (SRX 7.0.00rc07, Bruker, Germany). Lateral shift caused by the exchange was corrected either manually or using microspheres (TetraSpeck 0.1 μm, USA) as fiducial markers. For this purpose, microspheres were diluted to 1:500 in PBS, sonicated for 10 min, and added to the chambers. After settlement for 5 min, samples were washed thrice with PBS. For the microfluidic assisted exchange of labels in two-color and six-target STED imaging, a flow rate of 600 μL/min and total volumes of 1 mL (fluorescent label) and 2.5–5 mL (PBS) were used for labeling and washing, respectively. In total, exchange of fluorescent labels in the specimen using the microfluidic system was achieved within 5–9 min. In the case of manual pipetting, the sample was washed thrice using 150 μL of PBS with 2 min of incubation between washing steps, resulting in a total time of 6–8 min required for label exchange.

##### Simultaneous Two-Color Live-Cell STED Microscopy

For simultaneous two-color live-cell STED microscopy, Nile Red and JF_635_-S5 (xHTL) were diluted in LCIS to a final concentration of 500 nM and added to living cells seeded on microscopy chambers. After temperature adjustment, 30 consecutive frames were recorded in STED mode with an interval 17 s/frame. Detailed imaging settings are listed in Supplementary Table 4.

## Supporting Information Available

Supplementary Video 1: Single-color 3D-STED microscopy of the endoplasmic reticulum in U2OS cells labeled using xHTLs (MP4)
Supplementary Video 2: Two-color 3D-STED microscopy in a single cell using exchangeable fluorescent ligands (MP4)
Supplementary Video 3: Two-color live-cell STED microscopy using exchangeable fluorescent ligands (MP4)
Supplementary table on the imaging parameters used (Supplementary Table 4) (XLSX)
Experimental methods including chemical synthesis and image analysis; supplementary tables on antibodies, DNA-PAINT docking and imaging strands, xHTL ligands and PAINT labels used (Supplementary Tables 1–3); Supplementary Figures 1–4, Supplementary Spectrum 1 (PDF)

## Notes

A preprint of this work has been posted: GloggerM.; WangD.; KompaJ.; BalakrishnanA.; HiblotJ.; BarthH. D.; JohnssonK.; HeilemannM.Synergizing exchangeable fluorophore labels for multitarget STED microscopy. bioRxiv2022, 2022.07.02.498450. 10.1101/2022.07.02.498450PMC970678236223885

## References

[ref1] Hell S. W.; Wichmann J. Breaking the diffraction resolution limit by stimulated emission: stimulated-emission-depletion fluorescence microscopy. Opt. Lett. 1994, 19, 780–782. 10.1364/OL.19.000780. 19844443

[ref2] Heine J.; et al. Adaptive-illumination STED nanoscopy. Proc. Natl. Acad. Sci. U. S. A. 2017, 114, 9797–9802. 10.1073/pnas.1708304114. 28847959 PMC5604029

[ref3] Zheng Q.; Lavis L. D. Development of photostable fluorophores for molecular imaging. Curr. Opin. Chem. Biol. 2017, 39, 32–38. 10.1016/j.cbpa.2017.04.017. 28544971

[ref4] Danzl J. G.; et al. Coordinate-targeted fluorescence nanoscopy with multiple off states. Nat. Photonics 2016, 10, 122–128. 10.1038/nphoton.2015.266.

[ref5] Gonzalez Pisfil M.; et al. Triple-Color STED Nanoscopy: Sampling Absorption Spectra Differences for Efficient Linear Species Unmixing. J. Phys. Chem. B 2021, 125, 5694–5705. 10.1021/acs.jpcb.0c11390. 34048256

[ref6] Spahn C.; Grimm J. B.; Lavis L. D.; Lampe M.; Heilemann M. Whole-Cell, 3D, and Multicolor STED Imaging with Exchangeable Fluorophores. Nano Lett. 2019, 19, 500–505. 10.1021/acs.nanolett.8b04385. 30525682

[ref7] Spahn C.; et al. Protein-Specific, Multicolor and 3D STED Imaging in Cells with DNA-Labeled Antibodies. Ang. Chem. Int. Ed. 2019, 131, 19011–19014. 10.1002/ange.201910115. PMC6972974 31603612

[ref8] Perfilov M. M.; Gavrikov A. S.; Lukyanov K. A.; Mishin A. S. Transient fluorescence labeling: Low affinity—high benefits. Int. J. Mol. Sci. 2021, 22, 11799 10.3390/ijms222111799. 34769228 PMC8583718

[ref9] Sharonov A.; Hochstrasser R. M. Wide-field subdiffraction imaging by accumulated binding of diffusing probes. Proc. Natl. Acad. Sci. U. S. A. 2006, 103 (50), 18911–18916. 10.1073/pnas.0609643104. 17142314 PMC1748151

[ref10] Giannone G.; et al. Dynamic superresolution imaging of endogenous proteins on living cells at ultra-high density. Biophys. J. 2010, 99, 1303–1310. 10.1016/j.bpj.2010.06.005. 20713016 PMC2920718

[ref11] Jungmann R.; et al. Single-molecule kinetics and super-resolution microscopy by fluorescence imaging of transient binding on DNA origami. Nano Lett. 2010, 10, 4756–4761. 10.1021/nl103427w. 20957983

[ref12] Beater S.; Holzmeister P.; Lalkens B.; Tinnefeld P. Simple and aberration-free 4color-STED - multiplexing by transient binding. Opt. Express 2015, 23, 8630 10.1364/OE.23.008630. 25968701

[ref13] Wang Y.; et al. Rapid Sequential in Situ Multiplexing with DNA Exchange Imaging in Neuronal Cells and Tissues. Nano Lett. 2017, 17, 6131–6139. 10.1021/acs.nanolett.7b02716. 28933153 PMC5658129

[ref14] Kiuchi T.; Higuchi M.; Takamura A.; Maruoka M.; Watanabe N. Multitarget super-resolution microscopy with high-density labeling by exchangeable probes. Nat. Methods 2015, 12, 743–746. 10.1038/nmeth.3466. 26147917

[ref15] Eklund A. S.; Ganji M.; Gavins G.; Seitz O.; Jungmann R. Peptide-PAINT Super-Resolution Imaging Using Transient Coiled Coil Interactions. Nano Lett. 2020, 20, 6732–6737. 10.1021/acs.nanolett.0c02620. 32787168 PMC7496730

[ref16] Strauss S.; et al. Modified aptamers enable quantitative sub-10-nm cellular DNA-PAINT imaging. Nat. Methods 2018, 15, 685–688. 10.1038/s41592-018-0105-0. 30127504 PMC6345375

[ref17] Wilhelm J.; et al. Kinetic and Structural Characterization of the Self-Labeling Protein Tags HaloTag7, SNAP-tag, and CLIP-tag. Biochem. 2021, 60, 2560–2575. 10.1021/acs.biochem.1c00258. 34339177 PMC8388125

[ref18] Kompa J.; et al. Exchangeable HaloTag Ligands (xHTLs) for multi-modal super-resolution fluorescence microscopy. bioRxiv 2022, 2022.06.20.496706 10.1101/2022.06.20.496706.

[ref19] Spahn C. K.; et al. A toolbox for multiplexed super-resolution imaging of the E. coli nucleoid and membrane using novel PAINT labels. Sci. Rep. 2018, 8, 14768 10.1038/s41598-018-33052-3. 30282984 PMC6170473

[ref20] Ratz M.; Testa I.; Hell S. W.; Jakobs S. CRISPR/Cas9-mediated endogenous protein tagging for RESOLFT super-resolution microscopy of living human cells. Sci. Rep. 2015, 5, 9592 10.1038/srep09592. 25892259 PMC4402611

[ref21] Civitci F.; et al. Fast and multiplexed superresolution imaging with DNA-PAINT-ERS. Nat. Commun. 2020, 11, 4339 10.1038/s41467-020-18181-6. 32859909 PMC7455722

[ref22] Winheim S.; et al. Deconstructing the late phase of vimentin assembly by total internal reflection fluorescence microscopy (TIRFM). PLoS One 2011, 6, e19202 10.1371/journal.pone.0019202. 21544245 PMC3081349

[ref23] Culley S.; et al. Quantitative mapping and minimization of super-resolution optical imaging artifacts. Nat. Methods 2018, 15, 263–266. 10.1038/nmeth.4605. 29457791 PMC5884429

[ref24] Weber M.; et al. MINSTED fluorescence localization and nanoscopy. Nat. Photonics 2021, 15, 361–366. 10.1038/s41566-021-00774-2. 33953795 PMC7610723

[ref25] Puthukodan S.; Murtezi E.; Jacak J.; Klar T. A. Localization STED (LocSTED) microscopy with 15 nm resolution. Nanophotonics 2020, 9, 783–792. 10.1515/nanoph-2019-0398.

[ref26] Jahr W.; Velicky P.; Danzl J. G. Strategies to maximize performance in STimulated Emission Depletion (STED) nanoscopy of biological specimens. Methods 2020, 174, 27–41. 10.1016/j.ymeth.2019.07.019. 31344404 PMC7100895

[ref27] Kilian N.; et al. Assessing photodamage in live-cell STED microscopy. Nat. Methods 2018, 15, 755–756. 10.1038/s41592-018-0145-5. 30275592 PMC6915835

[ref28] Legant W. R.; et al. High density three-dimensional localization microscopy across large volumes. Nat. Methods 2016, 13, 359–365. 10.1038/nmeth.3797. 26950745 PMC4889433

[ref29] Benaissa H.; et al. Engineering of a fluorescent chemogenetic reporter with tunable color for advanced live-cell imaging. Nat. Commun. 2021, 12, 6989 10.1038/s41467-021-27334-0. 34848727 PMC8633346

[ref30] Torra J.; Bondia P.; Gutierrez-Erlandsson S.; Sot B.; Flors C. Long-term STED imaging of amyloid fibers with exchangeable Thioflavin T. Nanoscale 2020, 12, 15050–15053. 10.1039/D0NR02961K. 32666991

[ref31] Carravilla P.; et al. Long-term STED imaging of membrane packing and dynamics by exchangeable polarity-sensitive dyes. Biophys. Rep. 2021, 1, 100023 10.1016/j.bpr.2021.100023. PMC8651516 34939048

[ref32] Frei M. S.; et al. Engineered HaloTag variants for fluorescence lifetime multiplexing. Nat. Methods 2022, 19, 65–70. 10.1038/s41592-021-01341-x. 34916672 PMC8748199

[ref33] Winter F. R.; et al. Multicolour nanoscopy of fixed and living cells with a single STED beam and hyperspectral detection. Sci. Rep. 2017, 7, 46492 10.1038/srep46492. 28417977 PMC5394456

[ref34] Niehörster T.; et al. Multi-target spectrally resolved fluorescence lifetime imaging microscopy. Nat. Methods 2016, 13, 257–262. 10.1038/nmeth.3740. 26808668

