# Supplementary material for: Synergizing Exchangeable Fluorophore Labels for Multitarget STED Microscopy
Source: ACS Nano. 2022 Oct 12;16(11):17991–7. doi: 10.1021/acsnano.2c07212 (PMC9706782; doi:10.1021/acsnano.2c07212)
Supplement: Supplementary file 5 — nn2c07212_si_005.pdf [file nn2c07212_si_005.pdf]

Supplementary Information

Synergizing exchangeable fluorophore labels for multi-target STED microscopy

Marius Glogger[1]*, Dongni Wang[1], Julian Kompa[2], Ashwin Balakrishnan[1], Julien Hiblot[2], Hans-Dieter Barth[1], Kai Johnsson[2,3], Mike Heilemann[1]*

1 Institute of Physical and Theoretical Chemistry, Goethe University Frankfurt, Max-von-Laue Str. 7, 60438 Frankfurt, Germany

2 Department of Chemical Biology, Max Planck Institute for Medical Research, Jahnstrasse 29, 69120 Heidelberg, Germany

3 Institute of Chemical Sciences and Engineering (ISIC), École Polytechnique Fédérale de Lausanne (EPFL), 1015 Lausanne, Switzerland.

* Corresponding E-mail: glogger@chemie.uni-frankfurt.de; heilemann@chemie.uni-frankfurt.de

Table of contents

Experimental Methods 3
- Chemical Procedures 3
- Synthesis 4
- Image Analysis 8

Supplementary Tables **10**

Supplementary Figures 12

NMR spectra 16

Supplementary Videos **16**

References **17**

Experimental Methods

Chemical Procedures

All chemical reagents for synthesis were purchased from commercial suppliers (Acros, Fluka, Merck KGaA, Roth, Sigma-Aldrich, TCI, TOCRIS, AAT Bioquest) and used without further purification. Water-free solvents were stored over molecular sieves and used directly from a sealed-bottle. Reactions performed under air and moisture exclusion were carried out in heat-dried glassware and under inert argon atmosphere (Schlenk technique). Evaporation in vacuo was achieved using a rotary evaporator (Heidolph) or by lyophilization on a lyophilizer (Christ) equipped with a vacuum pump (Vacuubrand). Reaction progress was monitored by analytical thin-layer chromatography (TLC, POLYGRAM® SIL G/UV254, 0.2 mm layer pre-coated polyester sheet, 40 x 80 mm, Roth) or liquid chromatography coupled to mass spectrometry (LC-MS, Shimadzu LCMS2020 connected to a Nexera UHPLC system. Column: C18 1.7 µm, 50 × 2.1 mm (ACQUITY UPLC BEH, Waters). Buffer A: 0.1% Formic acid (FA)/MiliQ® water (ddH2O), buffer B: acetonitrile (MeCN). Typical gradient was from 10% to 90% B within 6 min with 0.5 mL min-1 flow.

Flash column purification was performed using a Biotage (IsoleraTM One) flash system equipped with pre-packed SiO2 columns (SiliaSepTM Flash Cartridges, 40 – 63 µm, 60 Å). Preparative reversed-phase high-performance liquid chromatography (RP-HPLC) was carried out on an UltiMate 3000 system (Thermo Fisher Scientific). Column: C18 5 µm, 21.2 × 250 mm (Supelco). Buffer A: 0.1% TFA in MiliQ® water, buffer B: MeCN. Typical gradient was from 20% to 90% B within 45 min with 8 mL min-1 flow. A 2998 PDA detector allows automated product collection based on the absorption wavelength of fluorescent labels (at 280, 550, 620 or 650 nm, respectively). 1H-, 13C- and 19F-NMR spectra were recorded in deuterated solvents on a Bruker Avance III HD 400 NMR spectrometer at 298 K at 400 MHz (1H), 101 MHz (13C) or 377 MHz (19F), respectively. Chemical shifts are expressed as parts per million (ppm, δ) and referenced to the solvent signals (1H / 13C) as internal standards: CDCl3 (7.26 / 77.16 ppm), CD3OD (3.31 / 49.00 ppm), DMSO-d6 (2.05 / 39.52) and MeCN-d3 (1.94 / 118.26 ppm) while 19F-signals were not referenced. Coupling constants J are reported in Hz. Signal descriptions include: s = singlet, d = doublet, t = triplet, q = quartet, p =pentet, s – sextet, h – heptet and m = multiplet. HRMS validation of synthesized chemical compounds was performed on a Bruker maXis IITM ETD spectrometer with electron spray ionization (ESI) by the Mass Spectrometry facility (MPImR, Heidelberg).

Synthesis

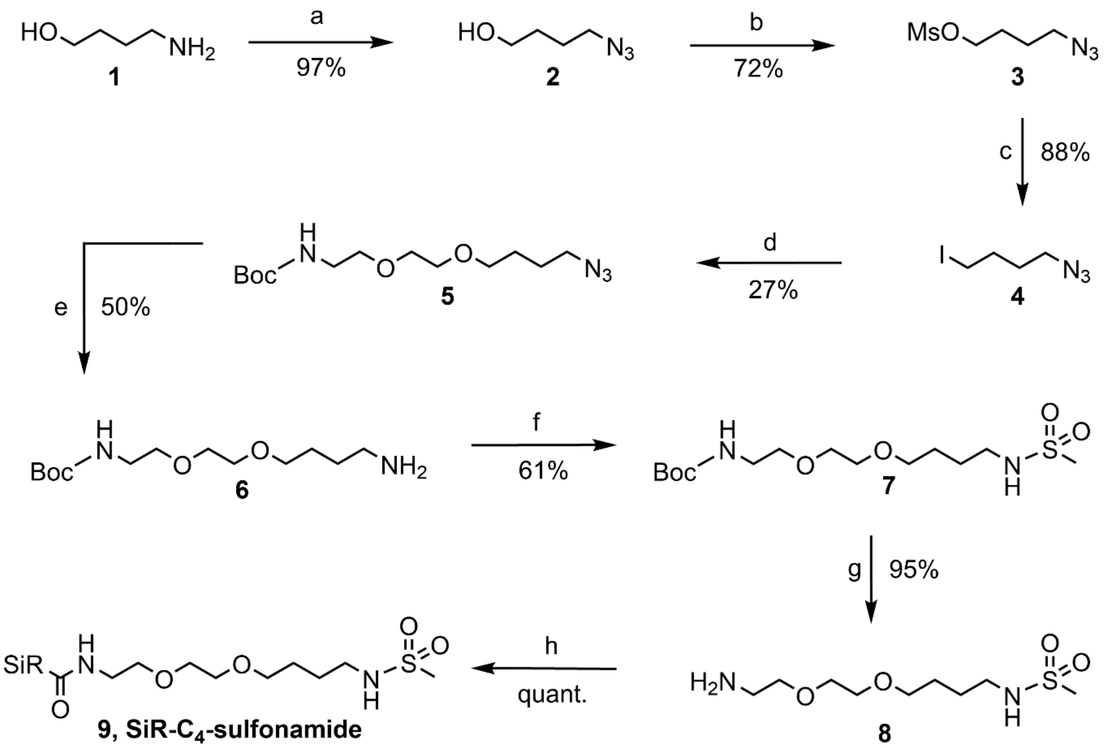

Supplementary Method Figure 1. Synthetic route to xHTL precursor Boc-PEG2-C4-methylsulfonamide (7). a. Imidazole-1-sulfonyl azide · HCl, K_2CO_3, Cu(II)SO_4 · 5 H_2O, MeOH, 12 h, 0 °C to room temperature (rt). b. MsCl, NEt_3, DCM, 3 h, 0 °C to rt. c. NaI, acetone, o/n, rt. d. BocNH-PEG2-OH, NaH, THF/DMF, 3 h, 0 °C to rt. e. PPh_3, THF/H_2O, 48 h, rt f. MsCl or TfCl, NEt_3, DCM, 3 h, 0 °C to rt. Boc: COO-C(CH3)3. g. TFA/DCM 1/1, 3 h, rt. h. SiR-COOH, TSTU, DMSO, DIPEA, 2 h, rt.

SiR-C4-sulfonamide synthesis

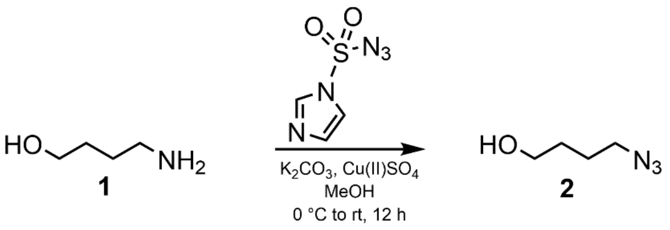

4-Azido-1-pentanol (2): A suspension of 4-amino-1-butanol (**1**, 1.2 mL, 13 mmol, 1 eq.), K_2CO_3 (4.1 g, 29.1 mmol, 2.25 eq.) and Cu(II)SO_4 · 5 H_2O (32 mg, 0.13 mmol, 1 mol%) was prepared in a heat-dried Schlenk-flask in 5 mL dry methanol (MeOH) under a stream of argon. The solution was cooled to 0 °C and imidazole-1-sulfonyl azide · HCl (3.3 g, 15.5 mmol, 1.2 eq.)[13] was added portion-wise. The mixture was left stirring for 12 h at rt, concentrated under reduced pressure and acidified with conc. HCl. The solid was taken up in 200 mL ethyl acetate (EtOAc), washed with 100 mL H_2O and brine, dried over $MgSO_4$, filtered and evaporated to afford the crude product as a pale-

yellow oil. Flash column chromatography (5 to 70% EtOAc in n-hexane for 10 CV) afforded compound **2** (1.6 g, 12.6 mmol, 97%) as a pale-yellow oil. **^{1}H NMR** (400 MHz, $CDCl_3$): δ 3.64 (t, *J* = 6.0 Hz, 2H), 3.30 (t, *J* = 6.5 Hz, 2H), 1.73 – 1.55 (m, 4H).**^{13}C NMR** (101 MHz, $CDCl_3$): δ 61.84, 51.16, 29.48, 25.20.

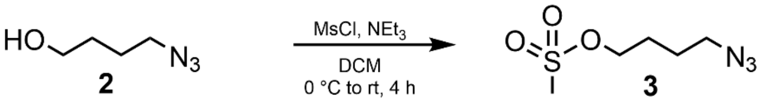

4-Azidobutyl-1-methansulfonate (3): A heat-dried round-bottom flask was charged with 65 mL dry DCM and triethylamine (NEt_3, 2.7 mL,19.5 mmol, 1.5 eq.). **2** (1.5 g, 12.6 mmol, 1 eq.) was added and the mixture was cooled to 0 °C. Methanesulfonylchloride (1.26 mL, 16.3 mmol, 1.25 eq.) was added dropwise under continued stirring and the solution was left stirring for 1 h at 0 °C, was warmed to rt and stirred for 3 h. 10 mL 10% NH_4Cl solution was added and the aq. layer was extracted three times with 100 mL DCM. The combined organic phases were washed with 50 mL brine, dried over $MgSO_4$, filtered and the solvent was removed. Flash column chromatography (0 to 3% MeOH in DCM for 9 CV) afforded compound **3** (1.8 g, 9.4 mmol, 72%) as a pale-yellow oil. **^{1}H NMR** (400 MHz, $CDCl_3$): δ 4.20 (t, *J* = 6.2 Hz, 2H), 3.29 (t, *J* = 6.6 Hz, 2H), 2.95 (s, 3H), 1.85 – 1.73 (m, 2H), 1.73 – 1.60 (m, 2H). **^{13}C NMR** (101 MHz, $CDCl_3$): δ 69.28, 50.37, 37.37, 26.33, 25.06. **HRMS** (m/z): $[M + H]^+$ calcd. for $C_5H_{11}N_3O_3SNa^+$, 216.0413; found, 216.0413

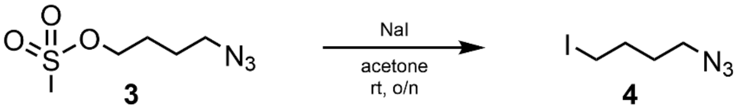

4-azido-1-iodobutan (**4**): **3** (1.8 g, 9.4 mmol, 1 eq.) and NaI (14 g, 94 mmol, 10 eq.) were mixed in 15 mL acetone in a round-bottom flask. The suspension was stirred at rt overnight. Residual solvent was removed *in vacuo* and the remaining solid was dissolved in 100 mL DCM and H_2O each. The aq. layer was extracted twice with 100 mL DCM. The organic layers were combined, washed with 100 mL sat. $Na_2S_2O_3$ and brine, dried over $MgSO_4$, filtered and the solvent was removed. Flash column chromatography (5 to 30% EtOAc in n-hexane in 6 CV) afforded compound **4** (1.8 g, 8.2 mmol, 88%) as a colorless liquid. **^{1}H NMR** (400 MHz, $CDCl_3$): δ 3.31 (q, *J* = 5.9, 5.3 Hz, 2H), 3.19 (q, *J* = 6.0, 5.4 Hz, 2H), 1.96 – 1.84 (m, 2H), 1.74 – 1.64 (m, 2H).**^{13}C NMR** (101 MHz, $CDCl_3$): δ 50.43, 29.81, 30.51, 5.83.

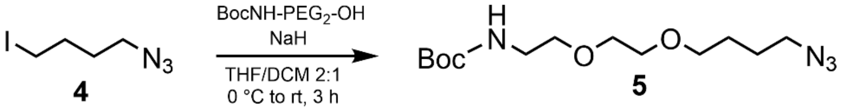

***tert*-Butyl N-[2-[2-(5-azidobutyloxy)ethoxy]ethyl]carbamate (5):** A heat-dried reaction tube was charged with 10 mL of a 2/1 ratio (v/v) of dry tetrahydrofuran (THF) and N,N-Dimethylformamid (DMF) under Schlenk

conditions. *tert*-Butyl(2-(2-hydroxyethoxy)ethyl)carbamate (BocNH-PEG$_2$-OH, 1 g, 4.8 mmol, 1 eq.) was added and dissolved under vigorous stirring. The mixture was cooled to 0 °C and NaH (220 mg, 60% immersion on mineral oil, 5.4 mmol, 1.1 eq.) was added portion-wise. The evolving gas was released carefully and the mixture was left stirring at 0 °C for 30 min under an inert gas atmosphere. **4** (1.6 g, 6.8 mmol, 1.4 eq.) was added directly into the suspension at 0 °C, warmed to rt and left stirring for 3 h. 10 mL 10% NH_4Cl and EtOAc were added and the aq. layer was extracted three times with 100 mL EtOAc. The org. layers were combined and washed with brine once and aq. 10% LiCl trice, dried over $MgSO_4$, filtered and the solvent was removed. Flash column chromatography (SiO_2. 20 to 50% EtOAc in n-hexane in 8 CV) afforded compound **5** (500 mg, 1.7 mmol, 27%) as a colorless oil. **^{1}H NMR** (400 MHz, $CDCl_3$): δ
4.99 (s, 1H), 3.63 – 3.44 (m, 8H), 3.30 (td, *J* = 6.6, 5.6, 3.0 Hz, 4H), 1.74 – 1.61 (m, 4H), 1.43 (s, 9H). **^{13}C NMR** (101 MHz, $CDCl_3$): δ 156.05, 79.24, 70.70, 70.28, 51.32, 40.36, 31.65, 28.50 (3C), 26.81, 25.80, 14.26**. HRMS** (m/z): [M + Na]$^+$ calcd. for $C_{13}H_{26}N_4O_4Na^+$, 325.1846; found, 325.1848.

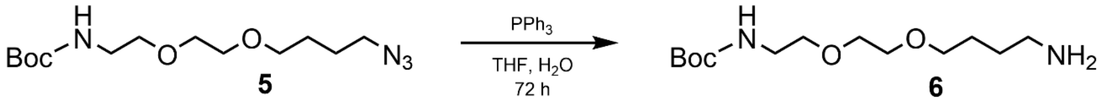

***tert*-Butyl N-[2-[2-(5-aminobutyloxy)ethoxy]ethyl]carbamate (6): 5** (550 mg, 1.5 mmol, 1 eq.) was dissolved in 2 dry THF in a dry round-bottom flask under an argon atmosphere. PPh_3 (550 mg, 2.3 mmol, 1.5 eq.) was added and the mixture was left stirring for 24 h air-excluded. Afterwards, H_2O (440 µL, 23 mmol, 15 eq.) were added and the mixture was left stirring for 48 h. The solution was concentrated *in vacuo* and flash column chromatography (SiO_2. 10% MeOH, 0.5% NEt_3 in DCM for 12 CV) afforded **6** (500 mg, 0.79 mmol, 50%) as a colorless liquid. **^{1}H NMR** (400 MHz, MeOD): δ 4.58 (s, 2H), 3.61 (s, 4H), 3.52 (dt, *J* = 14.8, 5.7 Hz, 4H), 3.22 (t, *J* = 5.7 Hz, 2H), 2.97 (t, *J* = 7.0 Hz, 2H), 1.83 – 1.65 (m, 4H), 1.44 (s, 9H). **^{13}C NMR** (101 MHz, MeOD): δ 157.30, 78.91, 70.27, 69.92, 69.86, 48.57, 49.92, 39.49, 27.47 (3C), 26.51, 24.85. **HRMS** (m/z): [M + H]$^+$ calcd. for $C_{13}H_{29}N_2O_4^+$, 277.2122; found, 277.2119.

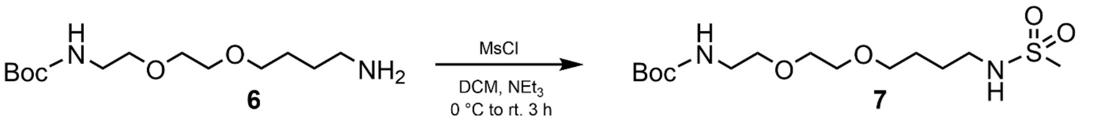

***tert*-Butyl (2-(2-((5-(methylsulfonamino)butyl)-oxy)ethoxy)ethylcarbamate (7)*.* 6** (100 mg, 0.34 mmol, 1 eq.) was dissolved in 2.5 mL dry DCM in a dry round-bottom flask. NEt_3 (70 µl, 0.51mmol, 1.5 eq.) was added and the mixture was cooled to 0 °C and stirred vigorously under an argon atmosphere. Methanesulfonyl chloride (32 µl, 0.42 mmol, 1.25 eq.) was added slowly at 0 °C. The mixture was stirred for 1 h at 0 °C, warmed to rt and left stirring overnight at rt. Afterwards, 5 mL DCM and 2.5 mL 1 N HCl was added and the aq. phase was extracted twice with 10 mL DCM. The combined organic layers were washed with 10 mL brine, dried over $MgSO_4$, filtered and the solvent was removed. Flash column chromatography (SiO_2. 20 to 80% EtOAc in n-hexane in 8 CV) afforded **7** (78 mg, 0.22 mmol, 61%) as a pale-yellow oil. **^{1}H NMR** (400

MHz, MeOD): δ 3.60 (s, 4H), 3.51 (td, J = 5.8, 4.3 Hz, 4H), 3.22 (t, J = 5.0 Hz, 2H), 3.08 (t, J = 6.5 Hz, 2H), 2.92 (s, 3H), 1.64 (tdd, J = 8.9, 3.9, 2.5 Hz, 4H), 1.44 (s, 9H). **^{13}C NMR** (101 MHz, $CDCl_3$): δ 157.06, 78.69, 70.40, 69.84, 69.77, 69.65, 42.45, 38.32, 29.37, 27.36 (3C), 26.55, 26.36. **HRMS** (m/z): $[M + Na]^+$ calcd. for $C_{15}H_{30}N_2O_6SNa^+$, 377.1717; found, 377.1714.

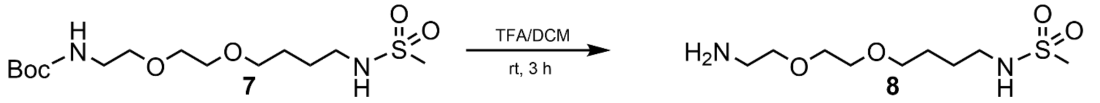

N-(4-(2-(2-aminoethoxy)ethoxy)butyl)methanesulfonamide (8). 7 (78 mg, 0.22 mmol, 61%) was dissolved in 3 mL/mmol of a 1/1 (v/v) mixture of trifluoroacetic acid (TFA) in dry DCM and stirred at rt for at 3 h. The solvent was removed under a stream of argon gas. Residual TFA was removed by co-evaporation with DCM. **8** · TFA (81 mg, 0.22 mmol, 95%) was collected and used without further purification. **^{1}H NMR** (Spectrum 1, 400 MHz, MeOD): 3.64 – 3.48 (m, 6H), 3.43 (t, J = 6.1 Hz, 2H), 3.07 – 2.94 (m, 4H), 2.82 (s, 3H), 1.62 – 1.48 (m, 4H). **^{13}C NMR** (101 MHz, $CDCl_3$): δ 70.44, 69.69, 69.72, 66.72, 42.41, 39.29, 38.25, 26.57, 26.27. **HRMS** (m/z): $[M + H]^+$ calcd. for $C_9H_{23}N_2O_4S^+$, 255.1373; found, 255.1370.

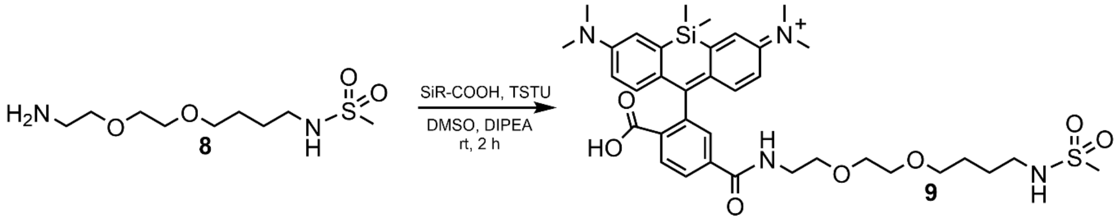

SiR-C$_4$-sulfonamide (SiR-S4) (9). 6-carboxy silicon rhodamine (SiR, 2 mg, 42 µmol, 1 eq.) was dissolved in 200 µL dry DMSO-d_6. The mixture was added on top of N,N,N′,N′-Tetramethyl-O-(N-succinimidyl)uroniumtetrafluorborat (TSTU, 1.5 mg, 51 µmol, 1.2 eq.) together with diisopropylethylamine (DIPEA, 70 µL, 420 µmol, 10 eq.) and left stirring at room-temperature. Meanwhile **8** (1.5 mg, 60 mmol, 1.4 eq.) was dissolved in the same amount of dry DMSO-d_6 with 10 eq. DIPEA. After 10 minutes both solutions were mixed and left stirring for 2 h at 40 °C. The desired products were obtained by RP-HPLC H_2O/MeCN linear gradient from 20 to 90% containing 0.1% TFA. **HRMS** (m/z): $[M + H]^+$ calcd. for $C_{36}H_{49}N_4O_7SSi$, 709.3086; found 709.3083.

Image Analysis

Intensity time traces of covalent and exchangeable HaloTag ligands

Intensity time-trace analysis of covalent and exchangeable HaloTag ligands was performed using a custom-written ImageJ macro (as described by Spahn *et al.*[1]). In brief, image sequences were drift-corrected using the ImageJ plugin "*StackReg*". User defined thresholding was then used to generate binary masks for image segmentation and extraction of signal and background intensity values. After correction for edge-effects, for each frame the signals from structures were averaged, background corrected and further analyzed in Origin software (Origin2019).

Determination of spatial resolution in STED images

To determine the spatial resolution in STED images, chemically fixed vimentin-HT7 expressing U2OS cells were labeled using xHTLs at concentrations of 100 nM and 500 nM. At high label densities, continuous signal was detected along the filaments, allowing the determination of the full-width half-maximum (FWHM) of the structure. For this purpose, a custom written analysis pipeline was established in python (https://github.com/MariusGlg/Filament_width_analyzer). In brief, 10-50 lines equally spaced and perpendicular to the vimentin filaments were drawn. Intensity profiles along the line segments (n = 691) were fitted with a Gaussian function and full-width half-maximum values (FWHM) were extracted for each segment. FWHM values were then plotted as a relative frequency distribution and fitted with a Gaussian function in Origin software. At low labeling densities, the point-spread-functions (PSF) of single vimentin-HT7 spots was analyzed as a measure of the spatial resolution. For this purpose, local intensity maxima of STED images reflecting the vimentin-HT7 positions were identified in ImageJ using intensity value thresholding ("*Find Maxima*" function) based on background and signal intensities. The ImageJ plugin "*GaussFit OnSpot*" was then used for fitting Gaussian profiles onto selected positions and extracting the FWHM (n = 88). As a third measure of the spatial resolution, the intensity profiles of vimentin structures at intersection points were analyzed. In brief, intensity line profiles perpendicular to the axes of vimentin filaments that were present in close proximity to each other were analyzed using ImageJ. The measured line profiles were then fitted using a multi-peak Gaussian function in Origin software to determine their full FWHM and mean. The FWHM and the distance between the means were taken to be the physical resolution achievable with biological samples in the setup used. In Fourier ring correlation based analysis of the image resolution, exchangeable and covalent HT7 labels were compared. For this purpose, chemically fixed cells were either covalently labeled with SiR-HTL (300 nM, incubation for 30 min at RT, followed by three washing steps) prior to imaging or directly imaged in 500 nM SiR-S5 (PBS). Following parameters were used for STED microscopy: 3% excitation power, 6% depletion laser power, 20 nm pixel size, pixel dwell time of 3 µs, 10x line accumulations and a pinhole size 0.71 AU. To create image pairs for the FRC analysis, the same ROI was imaged twice with no time delay between the imaging rounds to minimize sample drift. The FRC analysis was conducted using the NanoJ plugin in Fiji[2] with 10 measurements per condition and 20

blocks per image for good sampling of signal and background. Average FRC values per image were finally plotted in Origin software. For the determination of signal-to-noise and signal-to-background ratios, chemically fixed vimentin-HT7 expressing U2OS cells were prepared and imaged as described for the FRC analysis. The analysis was performed in Fiji. In brief, user defined intensity thresholds were used to create binary masks for the individual STED images. The mask was then applied to an image for segmentation of signal and background and mean pixel intensity values were then determined for both. Noise was determined as the standard deviation of the mean background pixel value in each image.

3D reconstruction of STED z-stacks

For volumetric rendering of STED z-stacks, images were first background subtracted in ImageJ using a rolling ball radius of 50 pixels. Images were then deconvoluted using the ImageJ plugin "*DeconWithGaussian*" and the z-position was color-coded using the plugin "*Z-stack Depth Colorcode*" (LUT spectrum). For single-color volumetric rendering and generation of 3D movies the plugin "*3D viewer*" was used. Dual-color 3D rendering and generation of dual-color movies was conducted in Napari open source software[3].

Supplementary Tables

Supplementary Table 1

Primary and secondary antibodies used for DNA-PAINT based labeling in this study.

Antibodies	Target	Dilution	Docking strands
Rabbit-anti-KDEL, abb176333, Abcam	Endoplasmic Reticulum	1:200	/
Rabbit-anti-TOM20, sc-11415, Santa Cruz	Mitochondria outer membrane	1:100	/
Mouse-anti-α-tubulin, T5168, Sigma-Aldrich	α-tubulin	1:200	/
AffiniPure donkey-anti-rabbit, #711-005-152, Jackson Immuno-Research	Rabbit primary antibodies	1:100	P1
AffiniPure goat-anti-mouse, #115-005-003, Jackson Immuno-Research	Mouse primary antibodies	1:150	P1
AffiniPure goat-anti-rabbit #111-005-003, Jackson Immuno-Research	Rabbit primary antibodies	1:150	P5

Supplementary Table 2

DNA docking and imager strands used in this study.

ssDNA oligo	Sequence	concentration (nM)
P1-docking strand	3´-GATC TAC ATA TT-5´-antibody	/
P1 imager strand	5´-TAG ATG TAT-3´-AbberiorSTAR$_{635P}$	300/500
P5 docking strand	3´-TAT GTA ACT TT-5´ -antibody	/
P5 imager strand	5´-C ATA CAT TGA-3´ -AlexaFluor$_{594}$<br>5´-C ATA CAT TGA-3´ -ATTO$_{655}$	300/500

Supplementary Table 3

xHTLs and PAINT labels for CLSM and STED microscopy.

Exchangeable label	Stock solution	concentration (nM)
JF$_{635}$-S5	100 µM (DMSO)	100, 300, 500
SiR-S4	100 µM (DMSO)	100, 300, 500
SiR-S5	100 µM (DMSO)	300
JF$_{646}$-Hoechst	100 µM (DMSO)	300
Nile Red	100 µg/mL	300/500
LifeAct-AF$_{594}$	1 mM (PBS, DTT, 10% glycerol)	1000
JF635-HTL	100 µM (DMSO)	100

Supplementary Table 4

Supplementary Table 4 contains a detailed overview of all CLSM and STED imaging parameters and is provided as a separate .xlsx file.

Supplementary Figures

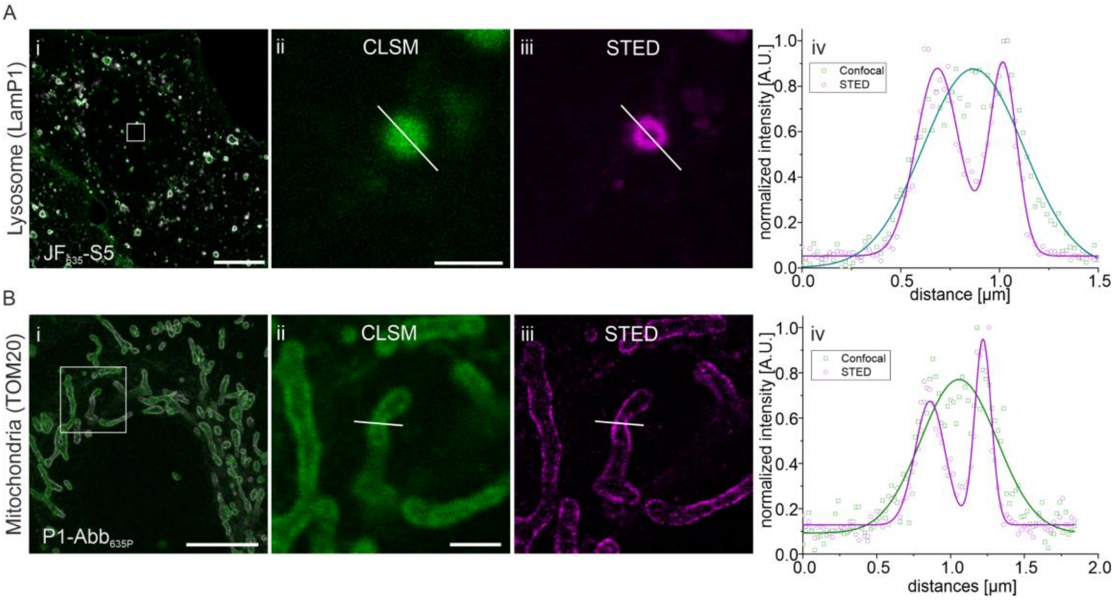

Supplementary Figure 1. Confocal and STED microscopy of lysosomes and mitochondria using exchangeable fluorophore labels. A) Confocal laser scanning microscopy (CLSM, green) and STED (magenta) image of lysosomes (LamP1-HT7, transient transfection) labelled using xHTLs (JF_{635}-S5) in a U2OS cell. i) Overview composite image with white box indicating a region of a single lysosome magnified in ii) and iii). iv) Normalized intensity profile across the lysosome (marked as white lines in ii) and iii)). The intensity distribution was fitted with a single (green) or two Gaussian functions (magenta). Scale bars are 10 µm (i) and 1 µm (ii-iii). B) CLSM (green) and STED (magenta) image of mitochondria (TOM20) labelled via DNA-PAINT (P1-$Abb_{635\,P}$) in a U2OS cell. i) Overview composite image with white box indicating a region magnified in ii) and iii). iv) Normalized intensity profile across single mitochondria (marked as white lines in ii) and iii). The intensity distribution was fitted with a single (green) or multi-gaussian function (magenta). Scale bars are 10 µm (i) and 2 µm (ii-iii).

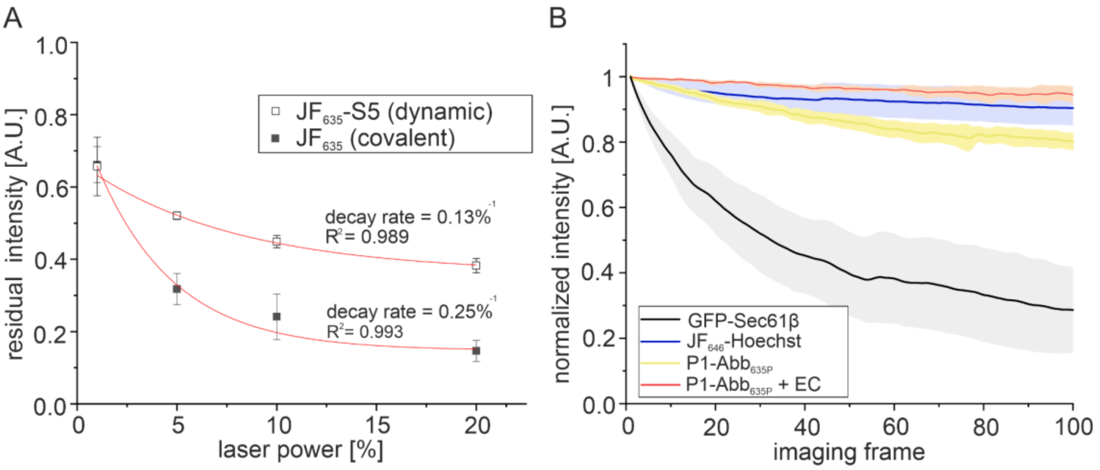

Supplementary Figure 2. Photostability characterization of different labelling approaches. Intensity over time trace for covalent versus transient labels (xHTLs) using confocal laser scanning microscopy. A) Quantitative analysis of the residual intensity after 25 consecutive frames and decay rates in U2OS cells expressing vimentin-HT7 and labelled with xHTLs. Comparison of covalent (JF_{635}-HTL) and transient (JF_{635}-S5) labels with increasing laser power (1 - 20 %). Decay rates were determined by fitting the residual intensity values with a mono-exponential decay function. Shown are mean values (N_{cells} = 5) ± standard deviations. B) Intensity-time trace analysis of covalent and transient labels over 100 frames at a laser intensity of 2 %. As a covalent label the ER-located fusion construct GFP-Sec61b was constitutively expressed in U2OS (grey line) and irradiated with 488 nm. For DNA-PAINT based exchangeable labels, the ER of U2OS cells was labelled with primary antibodies against KDEL and custom DNA-docking strand labelled secondary antibodies. Confocal imaging was performed using the imager strand P1-$Abb_{635\ P}$ (yellow line) at a concentration of 100 nM in PBS supplemented with 500 mM NaCl (pH 8,3). Kinetic tuning of the imager strand P1-$Abb_{635\ P}$ (red line) strand was achieved by the addition of ethylene carbonate (5% (w/v)) to the imaging buffer. As an exchangeable PAINT label the chromosomal DNA stain JF_{646}-Hoechst (blue line) was used at a concentration of 300 nM in PBS. All images were acquired at the Leica TCS SP8 confocal microscope. Shown are mean values (N_{cells} = 10) ± standard deviations. For the analysis of the intensity-time traces, a binary mask was applied to drift-corrected time-series and signal was segmented from the background. After background subtraction, intensity values were normalized with respect to the highest value.

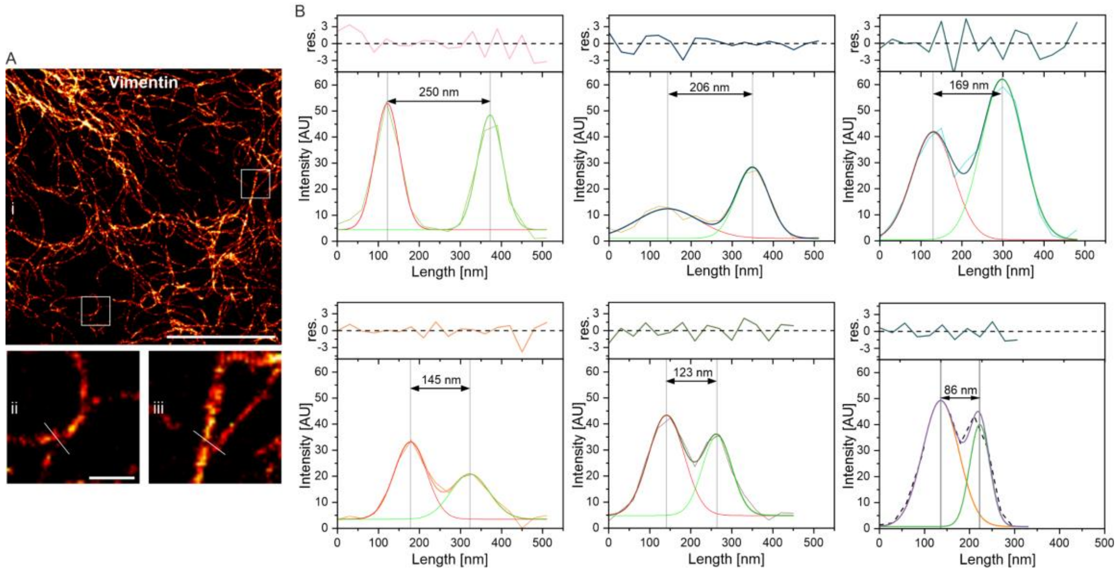

Supplementary Figure 3. Determination of the spatial resolution in STED images using vimentin intersection points. A) Super-resolved STED microscopy image of a U2OS cell expressing HaloTag7 conjugated to vimentin (vimentin-HT7). Vimentin-HT7 was labelled with the xHTL SiR-S5 (500 nM in PBS). ii – iii) Expanded views of the inlays shown as white boxes in (i). White lines indicate vimentin intersection points. Scale bars are 5 µm (overview) and 500 nm (magnified regions). B) Representative intensity distributions of various positions of two vimentin intersection points. Individual intensity distributions were fitted with a gaussian function and presented with residuals of the fit. The average FWHM of vimentin intensity distributions analyzed is 76 ± 20 nm (mean ± s.d.). The narrowest center-to-center distances of vimentin at intersection points (86 nm) reflects the minimal achievable resolution in the STED images.

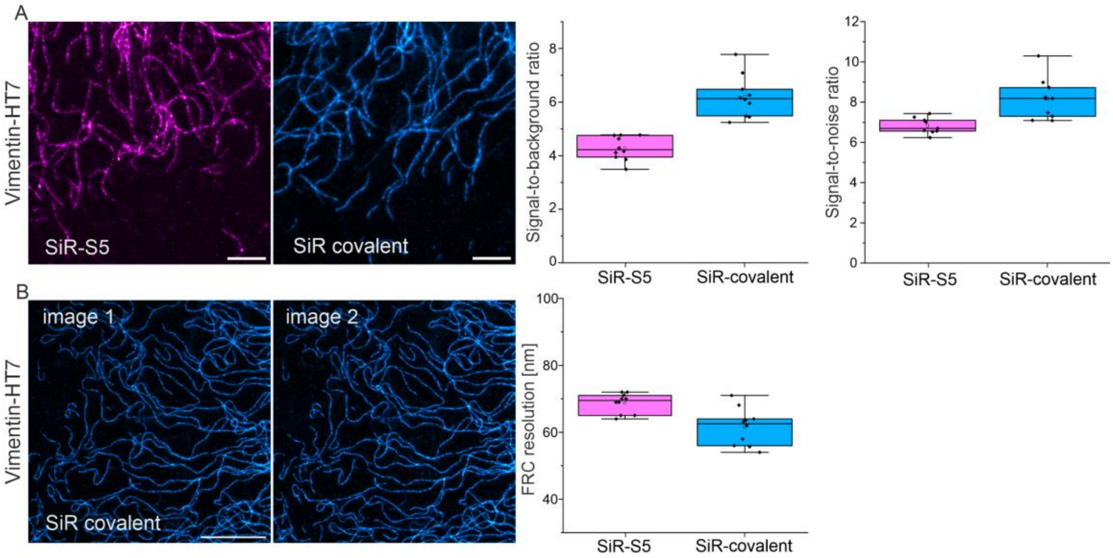

Supplementary Figure 4. Determination of Signal to noise ratio (SNR), signal to background ratio (SBR) and Fourier ring correlation (FRC) based image resolution in STED images. U2OS cell expressing HaloTag7 conjugated to vimentin (vimentin-HT7) and labeled with exchangeable (SiR-S5, 500 nM) and covalent (SiR covalent) fluorescent ligands. A. STED microscopy images of vimentin-HT7 labeled with SiR-S5 (magenta) and SiR covalent (cyan) ligands and their corresponding SBR and SNR (n_{images} = 10). B. Example of two tandem STED microscopy images (left) of vimentin-HT7 labeled with SiR covalent ligands and acquired with similar imaging settings for FRC analysis. right: FRC analysis of STED microscopy images of vimentin-HT7 labeled with SiR-S5 (magenta) and SiR covalent (cyan) ligands.

NMR spectra

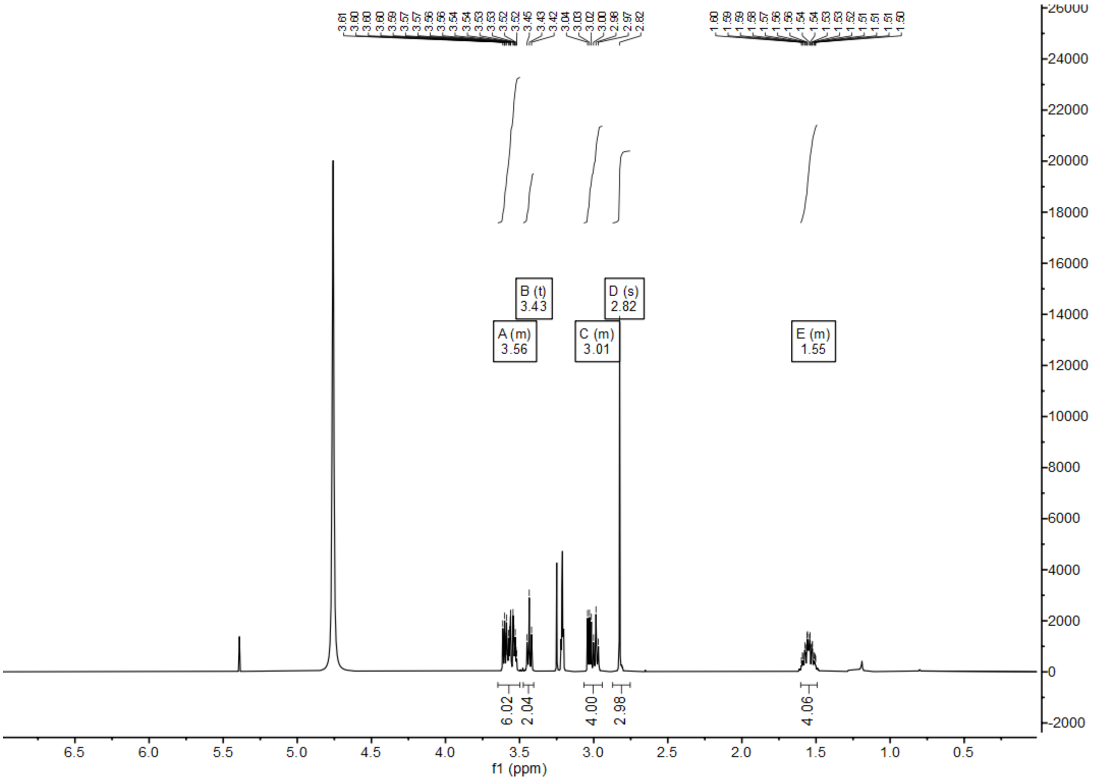

Supplementary Spectrum 1. ^{1}H-NMR (MeOD) of H_2N-NH-PEG_2-C_4-NH-SO_2-CH_3 (**8**).

Supplementary Videos

Supplementary_Video_1.mp4

Title: Single-color 3D-STED microscopy of the endoplasmic reticulum in U2OS cells labelled using xHTLs.

Legend: 3D-STED image acquisition and volumetric rendering of U2OS cells labelled for the ER-located fusion proteins CalR-HT7-KDEL using the exchangeable HT7 ligand SiR-S5 (300 nM). The axial position is color-coded (range 6 µm) using "spectrum" LUT.

Supplementary_Video_2.mp4

Title: 2-color 3D-STED microscopy in a single cell using exchangeable fluorescent ligands.

Legend: U2OS labelled for the ER (CalR-HT7-KDEL) and chromosomal DNA using the exchangeable ligands SiR-S5 (300 nM, magenta hot) and JF_{646}-Hoechst (300 nM, blue), respectively. Scale bar is 5 µm.

Supplementary_Video_3.mp4

Title: 2-color live-cell STED microscopy using exchangeable fluorescent ligands

Legend: Live U2OS cells labelled for vimentin (vimentin-HT7, orange hot) and cellular membranes (light blue) using the exchangeable fluorescent labels JF_{635}-S5 (500 nM) and Nile Red (500 nM), respectively. 30 consecutive frames were acquired with a framerate of 17 s.

References

1. Spahn, C. *et al.* Protein-Specific, Multicolor and 3D STED Imaging in Cells with DNA-Labeled Antibodies. *Angew. Chem. Int. Ed.* 131, 19011–19014 (2019).
2. Culley, S. *et al.* Quantitative mapping and minimization of super-resolution optical imaging artifacts. *Nat. Methods* **15**, 263–266 (2018).
3. Sofroniew, N. *et al.* Napari: multi-dimensional image viewer for python, https://github.com/napari/napari (2019).
